# The Role of Wearable Sensors in the Future Primary Healthcare – Preferences of the Adult Swiss Population: A Mixed Methods Approach

**DOI:** 10.1007/s10916-023-01998-1

**Published:** 2023-11-01

**Authors:** Corinne Matti, Stefan Essig, Zora Föhn, Andreas Balthasar

**Affiliations:** 1 Department Health Sciences and Medicine, University Lucerne, Lucerne, 6002 Switzerland; 2 Institute of Social and Preventive Medicine, University Bern, Mittelstrasse 43, Bern, 3012 Switzerland; 3 Interface Politikstudien Forschung Beratung AG, Seidenhofstrasse 12, Lucerne, 6003 Switzerland

**Keywords:** Wearable sensors, Wearable patient monitoring systems, Remote health monitoring, Population preferences

## Abstract

Wearable sensors have the potential to increase continuity of care and reduce healthcare expenditure. The user concerns and preferences regarding wearable sensors are the least addressed topic in related literature. Therefore, this study aimed first, to examine the preferences of the adult Swiss population regarding the use of wearable sensors in primary healthcare. Second, the study aimed to explain and learn more about these preferences and why such wearable sensors would or would not be used. An explanatory sequential design was used to reach the two aims. In the initial quantitative phase preferences of a nationwide survey were analyzed descriptively and a multivariable ordered logistic regression was used to identify key characteristics, that influence the preferences. In the second phase, eight semi-structured interviews were conducted. The cleaned study sample of the survey included 687 participants, 46% of whom gave a positive rating regarding the use of wearable sensors. In contrast, 44% gave a negative rating and 10% were neutral. The interviews showed that sensors should be small, not flashy and be compatible with everyday activities. Individuals without a current health risk or existing chronic disease showed lower preferences for using wearable sensors, particularly because they fear losing control over their own body. In contrast, individuals with increased risk or with an existing chronic disease were more likely to use wearable sensors as they can increase the personal safety and provide real-time health information to physicians. Therefore, an important deciding factor for and against the use of wearable sensors seems to be the perceived personal susceptibility for potential health problems.

## Introduction

Remote health monitoring has the potential to increase continuity of care and to reduce healthcare cost by shortening medical interactions and reducing travel time [1, 2].

In the last decade wearable patient monitoring systems have shown increasing research interest especially for health monitoring in outpatient care settings [3, 4]. These systems involve wearable non-invasive sensors, which are according to Bergmann et al. [5] defined as “any system that is connected to the body and measures clinical relevant information”. Wearable sensors are used remotely to measure a variety of health factors such as electrocardiogram, electromyogram, heart rate, blood pressure, blood oxygen saturation, body temperature and posture [1, 4]. More recently, there have been further advances in non-invasive wearable biosensors that allow metabolic parameters like electrolytes, glucose, pH and lactic acid to be measured via fluids external to the skin [6]. Wearable sensors can be integrated into smartwatches, armbands, rings, textile fiber, cloths or directly attached on the body (patches) [1, 7, 8].

The opportunities of such wearable sensors in the ambulatory setting include the early detection of emergency conditions and diseases such as cardiovascular, neurological and pulmonary diseases [1, 9, 10]. In addition, these sensors are useful for the continuous management of existing chronic conditions [4, 10]. To make use of these opportunities and the wearable patient monitoring systems, the wearable sensors must be connected to a smartphone via a near-field communication. The smartphone transmits the information to a cloud for data processing, which is commonly located at a healthcare facility. When the data processing indicates an abnormal condition or changes in physiological parameters, warnings are typically sent back to the user’s smartphone [4, 6, 11].

Whether individuals take advantage of wearable sensors depends on their willingness to change their health behavior [12]. According to the Health Belief Model (HBM) taking action to prevent or control a disease depends on the susceptibility and severity of the diseases as well as the benefits and barriers to a behavior [12]. The HBM helps to predict health behavior and has already been applied to explain health-preventative behavior such as vaccination against diseases [13].

Furthermore, acceptability of wearable sensors can be a driving force, but in its absence, it can also be a major barrier to wider adoption [14]. A systematic and scoping review regarding wearable technology for health monitoring showed that users’ concerns and preferences are the least addressed topic covered in related literature [10, 15]. Wang et al. [14] investigated consumer acceptance of wearable sensors according the Theory of Acceptance and Usage of Technology (UTAUT). The UTAUT is recognized as one of the most comprehensive theories to understand technology acceptance [16]. Their findings showed that an intention to use a wearable sensor is largely influenced by the users’ perceptions of the technology, meaning how consumers feel about a certain technology [14]. Furthermore, they showed that not only the perception but also technology characteristics have an influence on the intention to use a wearable sensor [14]. Previous established consumer preferences regarding wearable sensors include small, discrete and unobtrusive sensors, as they can be worn for 24 h a day [5, 17]. Data security and the potential loss of information, as well as cost, are other factors influencing the willingness to use wearable sensors [15, 17–19]. In order to increase the acceptance of wearable sensors user preferences should be further explored [5]. Moreover, these preferences are valuable for the design of such sensors to enable faster adoption in the primary care setting [4].

The technological and digital change are part of the Swiss health policy strategy 2020–2030. Switzerland is aware of technological opportunities for disease prevention and early detection, as well as for disease management [20]. Nevertheless, until the year 2020 little was known about the preferences of the adult Swiss population regarding wearable sensors in the primary healthcare setting. The research group of the project “Health2040” conducted a nationwide survey in Switzerland on preferences regarding future primary healthcare in outpatient settings [21]. With the partial utilization of the collected data, this study aimed first to investigate the preferences of the adult Swiss population regarding wearable sensors. Second, the study aimed to explain and learn more about these preferences and why wearable sensors would or would not be used.

## Methods

### Study design

An explanatory sequential design was chosen to analyze and explain the preferences regarding the use of wearable sensors in the future primary healthcare. This design involves two distinct phases, a quantitative and qualitative phase. Phase one represents the quantitative phase, based on the previous mentioned representative study. In phase two semi-structured interviews were conducted to explain the preferences identified in the quantitative phase.

### Participants

#### Quantitative phase

Participants for the quantitative survey were recruited via the sampling frame of the personal and household survey from the Federal Office of Statistics (N = 12,097) [21]. Every third invited participant was randomly asked about their preferences of wearable sensors, which gave a total of 4,026 individuals [21]. In total, 706 participants answered the relevant question for this study (356 male & 349 female).

#### Qualitative phase

As participants for the first phase were randomly and anonymously selected it was not possible to select participants for the qualitative phase from the same sample frame. A purposive sampling strategy was used to find appropriate participants for the qualitative phase. This refers to a selective sampling of participants based on characteristics of interest. Results of the quantitative phase indicated that the main characteristic of interest for the qualitative phase was health status. Ultimately, four participants with and without a chronic disease were interviewed (n = 8). After eight interviews, data saturation was considered to be achieved as no new ideas or categories appeared in the analysis.

### Data collection

#### Quantitative phase

Individuals were invited to participate in the online survey by mail [21]. The question of interest for this study included a short description of a scenario. Participants should imagine wearing a sensor that measures several physiological parameters, which then are transferred to their primary care physician. The information is used to detect potential health complications, such as a heart attack. If complications are detected, the user receives a notification on the smartphone recommending a visit to the doctor. Participants were asked to rate the scenario based on a 7-point Likert scale. The answer options range from “Don’t like it at all”, “Don’t like it”, “Tend to not like it”, “Neither like nor dislike it” to “Tend to like it”, “Like it” and “Like it a lot”.

#### Qualitative phase

Before the scheduled interviews, participants received a brief description of two scenarios to introduce the topic. Scenario one was the same as used in the survey. Based on the findings of the quantitative phase, which indicated that the health status is an important factor influencing preferences, a second scenario was developed. In scenario two, the wearable sensor user has an existing chronic disease and the sensor is intended for disease management. As before, physiologically relevant parameters are measured and forwarded to the primary care physician. If complications or a deterioration of the chronic disease are detected, the user receives a notification with the same recommendation as before. The interviews were conducted via Zoom or over the telephone. An interview guide was developed and pre-tested. Questions are based on the two presented scenarios and findings of previous studies [5, 15, 17] to investigate preferences to use wearable sensors and important features related to their use (see Appendix A.1). To investigate whether there are differences between the two scenarios, participants answered the questions by first imagining themselves in scenario one and then two.

### Data analysis

#### Quantitative phase

The dataset was entered to the statistical software Stata (Version 16.1) for data cleaning and analysis. After excluding those participants with missing information (n = 19) the preferences for wearable sensors were analyzed descriptively for the remaining 687 participants. The answer options of the Likert scale were categorized as following: “Like it a lot”, “Like it” and “Tend to like it” are evaluated as positive ratings. Whereases “Don’t like it at all”, “Don’t Like it” and “Tend to not like it” are assessed as negative ratings. “Neither like nor dislike it” represents the neutral option. Second, a multivariable ordered logistic regression was used to identify demographic and sociodemographic characteristics independently associated with the preference for wearable sensors. The output of the regression indicates the odds of moving to a more positive preference category for each investigated variable. In line with other studies [22, 23] on patient preferences most common demographic and sociodemographic characteristics namely sex, age, education status, place of residence and health status were included for the statistical analysis. The variable sex was divided into female and male whereas age was included as continuous variable. Education status was categorized into tertiary and up to secondary education as only very few participants had a primary education. The variable place of residence contained two categories sub-urban / urban and rural, and health status was defined as not chronically ill and chronically ill.

#### Qualitative phase

The audio recorded interviews were transcribed in form of intelligent verbatim [24, 25]. The data analysis was based on Braun and Clark’s thematic analysis [26]. The first phase involved reading and re-reading the transcripts to get familiar with the data. Second, initial codes of relevant information were generated. In a third step sub-themes, which link similar codes, were developed. Sub-themes of a certain topic were then linked to an overall theme. During this phase a thematic map with the identified themes and sub-themes was developed to illustrate the different categories and their relationship. Phase four included the reviewing of the generated themes. The final themes were named in phase five and in a final step the results were written down and the thematic map was finalized [26, 27].

## Results

### Phase 1: Quantitative findings

Table 1 below describes the investigated variables. Looking at the preferences for wearable sensors a total of 46% gave a positive rating. The negative ratings add up to 44% and 10% could not say whether they like or dislike the idea of wearable sensors.

**Table 1:** Participants demographic & sociodemographic characteristics

**Characteristics**	Survey	Interview
	(N = 687)	(N = 8)
	n (%)	
**Sex**		
Male	347 (50.5)	4 (50)
Female	340 (49.5)	4 (50)
**Age (years)**		
Mean (SD)	48.94 (16.5)	43.37 (19.1)
Range	19-90	24-73
**Education status**		
Up to secondary	371 (54)	4 (50)
Tertiary	316 (46)	4 (50)
**Place of residence**		
Rural	142 (20.7)	2 (25)
Sub-urban / Urban	545 (79.3)	6 (75)
**Health status**		
Chronically ill	240 (34.9)	4 (50)
Not chronically ill	447 (65.1)	4 (50)
**Preferences wearable sensor**		
Don’t like it at all	201 (29.3)	
Don’t like it	55 (8)	
Tend to not like it	43 (6.3)	
Neither like nor dislike it	71 (10.3)	
Tend to like it	108 (15.7)	
Like it	81 (11.8)	
Like it a lot	128 (18.6)	

In Table 2 the results of the multivariable ordered logistic regression are presented. The differences concerning preferences were insignificant for age, education level and place of residence. On the other hand, being female was negatively associated with liking wearable sensors (*P* < 0.05, OR 0.75). In addition, participants not suffering from a chronic disease had decreased odds to like wearable sensors (P < 0.1, OR 0.75). To put it in other words, male and chronically ill individuals had increased odds to be in a more positive preferences category compared with female and not chronically ill individuals.

**Table 2:** Ordered logistic regression

	**OR (95% CI)**
**Age (18–90)**	1.00 (0.96–1.05)
**Education** (Ref. up to secondary)	
Tertiary	0.86 (0.66–1.14)
**Place of residence** (Ref. rural)	
Sub-urban / Urban	1.20 (0.87–1.65)
**Sex** (Ref. men)	
Female	0.75 (0.57–0.98)**
**Health status** (Ref. chronically ill)	
Not chronically ill	0.75 (0.57-1.00)*

OR odds ratio, CI confidence interval, Ref. Reference category

*p < 0.1; **p < 0.05

### Phase 2: Qualitative findings

**Fig. 1:**
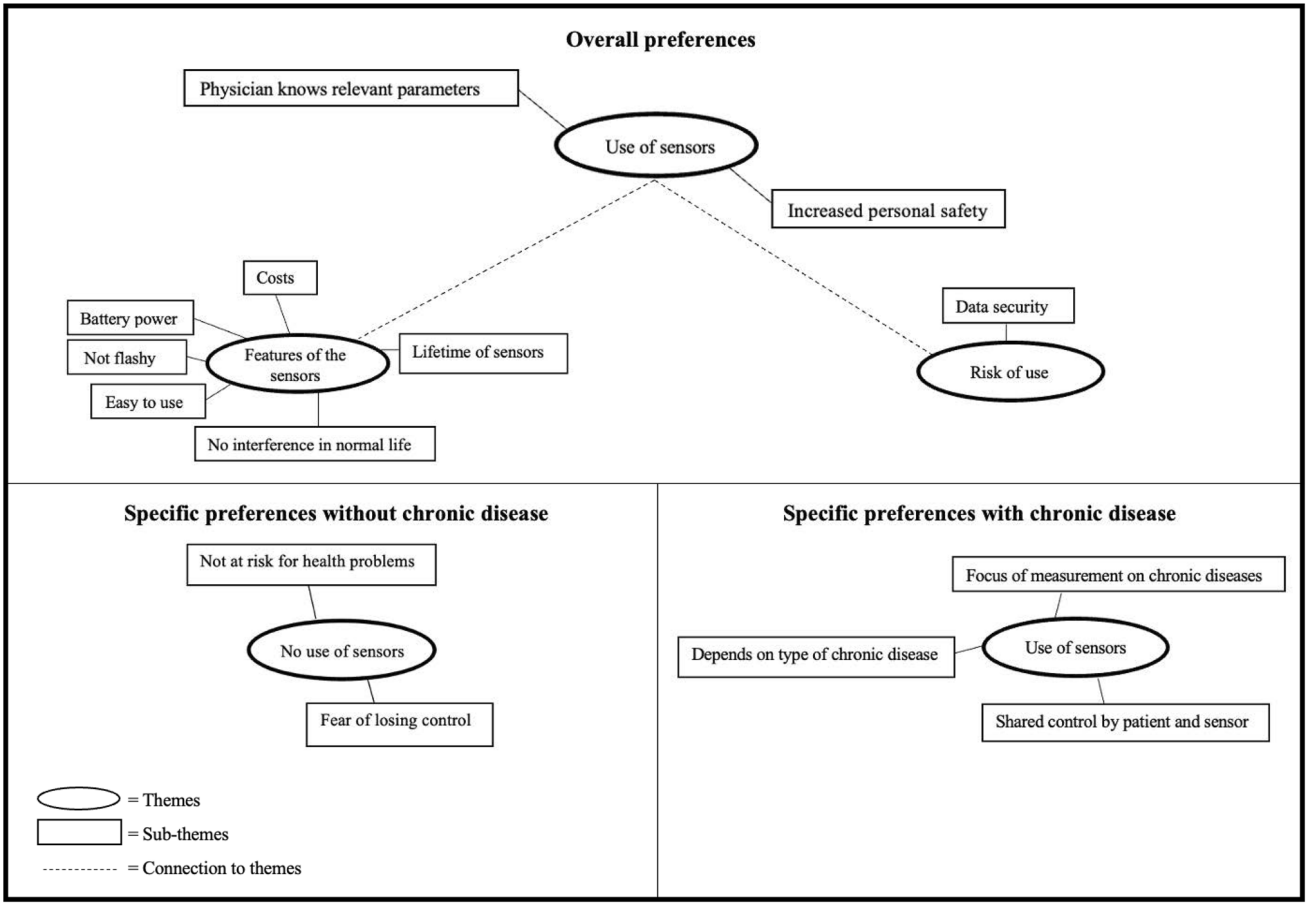
Thematic map

Through in-depth discussion with the interviewees various preferences were identified. Figure 1 illustrates a thematic map with the most relevant themes and corresponding sub-themes developed through the thematic analysis. First, the overall preferences including both scenarios are presented. Second, the preferences regarding scenario two (with chronic disease) are illustrated and in the last step the preferences regarding scenario one (without chronic disease) are discussed. The Appendix A.2 contains the complete thematic map followed by the corresponding explanations (Appendix A.3-A.9).

#### Overall preferences

This section first covers the theme “use of sensors” which includes reasons why interviewees would use wearable sensors presented in the two scenarios. Furthermore, the intention to use wearable sensors was associated with several necessary features, which are also introduced in this section. Finally certain risks in connection with the use of wearable sensors were established, which are highlighted as a last theme of this section.

#### Use of sensors

##### Increased personal safety

Increased personal safety was in most cases the first point mentioned when participants explained why they would use a wearable sensor. The biggest advantage was seen in the early detection of an emerging diseases, another acute health complications and the worsening of existing chronic diseases. For the interviewed persons the wearable sensor would provide reassurance by constantly measuring relevant health parameters. Deteriorations of vital parameters are not always directly noticeable and with the help of a wearable sensor, changes can be detected and treated at an early stage. Health predictions supported by algorithms and artificial intelligence are, according to some participants, better and faster than when performed by humans. The word that was often used to describe these benefits was “safety.“ Safety not only in the sense of early detection, but also the knowledge that someone will be informed and one is not left alone in emergency situations.

##### Physician knows relevant parameters

Another reason for using a wearable sensor would be that the primary care physician automatically has access to real-time information about a person’s health.

“Why should the doctor have to write all this down in his or her computer when he or she could actually have it already”.

This would save both the patient and the doctor valuable time. Ultimately, it would be an improvement and simplification for the healthcare system, as the patient would not have to go through multiple tests anymore.

#### Features of the sensors

##### Not flashy

The design of wearable sensors was an often discussed topic and appeared to be one of the most relevant features. According to the interviewees the sensor should be small and not flashy. The sensor should also not have a medical appearance. The reasons for these points were partly aesthetic and partly social. Participants do not want to be asked what they are wearing and why they are wearing it.

##### No interference in normal life

Not interfering with normal life was a further important feature frequently mentioned by participants. Sensors should be compatible with everyday activities such as sports, should not disturb during the night, should be resistant to water and cold weather.

##### Battery power

The preferences in terms of battery power were very similar. A wearable sensor needs to last at least a full day to provide 24-hour monitoring. Anything less than a day would be annoying and not worth buying for the interviewees. A week, or as long as possible would obviously be desirable in terms of battery life. Establishing a routine was an important point mentioned by some respondents. If it is not less than once a day, but always at the same time, it does not matter if the sensor needs to be charged once a day or once a week. Nevertheless, there were skeptical thoughts when the sensor had to be charged every day. It becomes difficult when one is not at home at night and no power source is available.

##### Easy to use

The handling of the sensor should be, according to the participants, self-explanatory, require few steps to use, and little effort to avoid permanent removal of the sensor. Some would like the sensor and associated instructions to be provided by the primary care physician. With clear guidance from the primary care physician regarding the exact use of the sensor, application errors can be minimized. In addition, different instructions should be provided for specific target groups. For example, the instructions should be available in different languages and include pictures if reading is not possible.

##### Lifetime of sensors

Before the cost component could be discussed, the lifetime of wearable sensors had to be determined first. Due to technical progress, the participants expected lifetime of a wearable sensor should be between three and five years.

##### Costs

When the cost component was introduced, everyone assumed that it would be covered by health insurance. First, to ensure that everyone has access to a wearable sensor if they want it, and second, because health insurance companies should be interested in preventive healthcare. If the costs were not covered by health insurance, interviewees would be willing to pay around 85–700 Swiss francs ($\overline{x}$: 270.-) per year for a wearable sensor. The willingness to pay was very high for some people, as they consider health to be something very valuable and worth paying for.

#### Risk of use

##### Data security

Interviewees all agreed that data security is a very important issue, but it was assessed differently. Some do not see any problems if their personal data gets disclosed and is used by external actors. They believe that their health parameters are not important to others. However, others see many potential harms if the data goes beyond the treating physician. External actors are enriching themselves with very personal data that can be used against the individual. The personal data could be used for tailored advertising, to exclude someone based on their health parameters, or it could cost someone’s job if health information gets to the employer. Although there is a certain risk of data misuse or breach, in the participants’ views, benefits of wearable sensors outweigh these risks.

#### Specific preferences with chronic diseases

This section focuses on the reasons why some interviewees would only use wearable sensors if they were chronically ill.

Use of sensors

##### Focus of measurement on chronic diseases

Those participants who are only interested in using a wearable sensor in the context of scenario two, explained that in such a situation it is clear which parameters need to be measured constantly. Individuals who are chronically ill have certain risk factors which need to be monitored to avoid complications.

##### Shared control by patient and sensor

Combined with the knowledge of important parameters to be measured, the use of a wearable sensor would allow patients to relinquish control. Since the measurement is limited to the relevant parameters, patients do not have to think about it all the time and can rely on the sensor.

##### Risk of life-threatening diseases

The use of wearable sensors would still be dependent on the type of chronic disease. The participants would only be willing to use a sensor if the chronic disease is fatal and life would likely be extended by its use. One chronic disease category for which a wearable sensor would be used, was a cardiovascular disease. In this case, the sensor could indicate early on when something is wrong, and premature death could be avoided.

#### Specific preferences without chronic disease

This section focuses on the reasons for not using wearable sensors in the absence of a chronic disease.

No use of sensors

##### Not at risk for health problems

Participants opposed to wearable sensors stated that the deciding factor for using a wearable sensor should be the risk assessment. In their perception, measuring various parameters in healthy individuals has no benefits as they are not at risk for health problems. Wearable sensors should only be used in individuals with a known risk, a hereditary predisposition or an existing chronic disease. The older generation in particular would benefit from wearable sensors, as they have multiple risk factors due to their age.

##### Fear of losing control

Furthermore, the use of wearable sensors is not necessary because the participants said they have a strong body awareness. They would notice when something is wrong and fear that using a sensor would take away this body awareness. They would lose the ability to notice changes in the body and relinquish control to the device. This decreased self-control would make them dependent on the wearable sensor and lead to decreased personal responsibility. Participants are unwilling to give up their personal responsibility and decision-making about their own bodies.

## Discussion

### Summary of findings

The aims of this study were to investigate and explain the preferences of the adult Swiss population regarding wearable sensors in primary healthcare using an explanatory sequential design. The main findings of the survey showed that there were slightly more positive ratings compared to negative, 46% and 44% respectively, with 10% unable to decide. A multivariable ordered logistic regression showed that the preferences were significantly influenced by health status and gender. Chronically ill and male individuals were more likely to show an increased preference regarding the use of wearable sensors. The semi-structured interviews provided detailed insight into the preferences and various reasons for and against the use of wearable sensors. Individuals without a current health risk or existing chronic disease showed lower preferences for using wearable sensors, particularly because they fear losing control over their own body. In contrast, preferences of using wearable sensors were increased for individuals with increased health risk or with an existing chronic, as these can increase the personal safety and provide real-time health information to physicians.

### Comparison of evidence

Compared to previous studies on consumer acceptance of wearable technology in healthcare similar preferences regarding features of the wearable sensors were identified in the underlying study [5, 17, 18]. The most important features for the interviewees were that sensors should be small, not flashy and not interfere in normal life. According to Bergmann et al. [5] the most important features of wearable sensors were to be comfortable and not to interfere with daily behavior, while discreteness was one of the least important features.

Also in line with the findings of this study is a survey study on consumers’ perceived attitude on wearable sensors, which showed the expected lifetime and costs of wearable sensors are four years and below 300 Swiss francs [18].

Data security and privacy requirements of wearable sensors are an often discussed topic in the literature [15, 17–19, 28, 29]. Privacy concerns and potential leakage of personal health information are according to Wen et al. [18] important factors affecting the acceptance of wearable sensors. Gao et al. [17] furthermore speak of a tradeoff between perceived benefits of the wearable sensor and perceived privacy risk when deciding whether to adopt a wearable sensor. The data security and privacy concerns were also perceived by the participants in the underlying study, but these risks did not exceed the potential benefits of wearable sensors. The established features support the findings of Wang et al. [14] which state that if certain characteristics, such as the appearance of a sensor, are given then consumers are more likely to accept it.

In contrast to another study where females showed greater preference for wearable technology this study revealed that females would less likely to use wearable sensors [30]. Whether differences in wearable sensors preference depends on gender remains unclear. The Health Belief Model could offer a possible explanation, independent of gender, for not using wearable sensors. The main reason respondents would not want to use wearable sensors was the lack of a current risk for a health threat. In other words, and in line with the model, respondents did not perceive themselves to be susceptible for health problems. Even if they were susceptible, the severity of the disease would still influence the decision for using wearable sensors. With the lack of perceived susceptibility for health problems individuals perceive no benefits by using wearable sensors and the loss of control over their own body is evaluated as major barrier. Therefore, individuals without a current health risk or an existing chronic disease showed decreased preferences for wearable sensors.

Based on UTAUT performance expectation, social influence, facilitating conditions and effort expectancy influence the technology acceptance [16]. According to Wang et al. [14] performance expectation is the most important determinant for the behavioral intention to use wearable sensors. Performance expectation refers to the extent to which individuals belief that a wearable sensor can help to reduce health-related threats. Interviewed individuals, who showed a lack of perceived susceptibility, had no performance expectations from a wearable sensor. On the other hand, interviewed individuals, who perceived themselves as susceptible for health problems, believed that wearable sensors can reduce health-related threats. For these individuals the other three determinants of the UTAUT also influenced their preferences. The social influence refers to the agreement of important others [14]. Accordingly, interviewees mentioned that wearable sensors should be small and not flashy to avoid other people asking about it. Facilitating conditions include the technical support in form of knowledge and skills [14]. Respondents indicated that instructions for the correct use should be provided by the primary care physician to avoid application errors. Effort expectancy describes the handling of wearable sensors, which should be easy and effortless [14]. Interviewees highlighted that the use of wearable sensors should be self-explanatory and require little effort to avoid permanent removal of the sensor.

## Strengths and limitations

The strengths of this underlying study lie in the use of a mixed methods approach. Combining quantitative and qualitative methods together led to an in-depth investigation and explanation of the preferences regarding wearable sensors. Nevertheless, the study has some limitations. As the quantitative results were based on a cross-sectional data collection the established relationships in the multivariable regression need to be treated with caution. In addition, the interview recruitment process may have influenced the results, as individuals interested in this field were more likely to participate. Furthermore, interviewees answers might have been affected by the presence of the interviewer and did not reflect their reality, although confidentiality and anonymity were assured.

## Implications

This is the first study that provides detailed insight into the preferences of the adult Swiss population regarding wearable sensors in the primary healthcare setting. The identified key features present a guideline for enhancing the design and functionality of wearable sensors. Furthermore, findings showed that determinants of the UTAUT framework indeed influence user preferences. By incorporating these features into future development, user acceptance and intention to use can be expected to increase, which will support an effective implementation into healthcare practices. With additional population-based quantitative studies precise factors that influence individuals’ preferences towards wearable sensors can be investigated in more depth. Profound insight into preferences will allow targeted strategies for implementation by tailoring it to the most suitable user population.

## Conclusion

This study provides valuable insights into the preferences of the adult population regarding wearable sensors in primary healthcare. Results indicate that the intention to use a wearable sensor depends on the perceived susceptibility for health threats. Individuals with an increased health risk or an existing chronic disease showed increased preferences of using wearable sensors in the future. Nevertheless, the acceptance of wearable sensors highly depends on their specific features. Sensors should be small, not flashy and not interfere with daily activities. Furthermore, technical support of a primary care physician and effortless handling should be guaranteed to enhance the intention to use wearable sensors.

## Appendix

### A.1 Interview guide

#### Introduction

- Do you regularly monitor any bodily functions? (Pulse, blood pressure, etc.)
- Do you own a sensor (e.g., a smartwatch)?
  - If yes, what do you use this sensor for?
    - For sports-related purposes.
    - For medical purposes.
  - If no, why don’t you have a sensor?
- How do you currently assess your state of health?
  - On a scale of 0 (very poor) to 10 (very good), where would you rate your state of health?

### Main part

#### Scenario 1

Imagine that in 2040, you consistently wear a sensor on your body. This sensor transmits data about your bodily functions to your primary care physician. The data is used for digitally monitoring your state of health in order to predict imminent complications, such as a heart attack or stroke. If such complications are detected, your smartphone will prompt you to visit a doctor.

#### Scenario 2

Imagine you have a chronic illness in 2040 and wear a sensor on your body. This sensor also measures and transmits data about your bodily functions to your primary care physician. The data is utilized for digitally monitoring your state of health. If your health condition deteriorates, your smartphone will prompt you to visit your doctor.

- What goes through your mind when you read these scenarios?
- In other words, would you consider using such a sensor?

If yes:

- Why would you use such a sensor?
- What do you like about it?
  - Why?
  - What are advantages?
- Under which circumstances would you use such a sensor?
  - Privacy/Confidentiality.
  - Accurate Measurement / Measurement Errors.
    - Consistency of Measurement.
  - Complexity/Simplicity (Connecting to Smartphone).
  - Battery (Efficient Usage).
  - Comfort while Wearing (Comfortable or Lightweight).
  - Communication Speed.
  - Costs.
    - If basic health insurance covered the costs, would you consider getting a sensor?
    - How much would you be willing to pay for such a sensor?
- If complications are detected, in your opinion, who should be informed?
  - Primary care physician?
  - Patient?
  - Both?
  - Others? (Emergency services, air rescue, family, etc.)
    - Please provide reasons for your answer.

If no:

- What don’t you like about such sensors?
  - What could change your mind?
- Under what circumstances could you envision using such a sensor?
  - Privacy/Confidentiality.
  - Accurate Measurement / Measurement Errors.
    - Consistency of Measurement.
  - Complexity/Simplicity (Connecting to Smartphone).
  - Battery (Efficient Usage).
  - Comfort while Wearing (Comfortable or Lightweight).
  - Communication Speed.
  - Costs.
    - If basic health insurance covered the costs, would you consider getting a sensor?
- If the circumstances you mentioned were guaranteed at 100%, would you now use a sensor?
  - Can you provide reasons for your answer?

#### Final question

- Is there anything else you would like to add?

### A.2 Complete thematic map


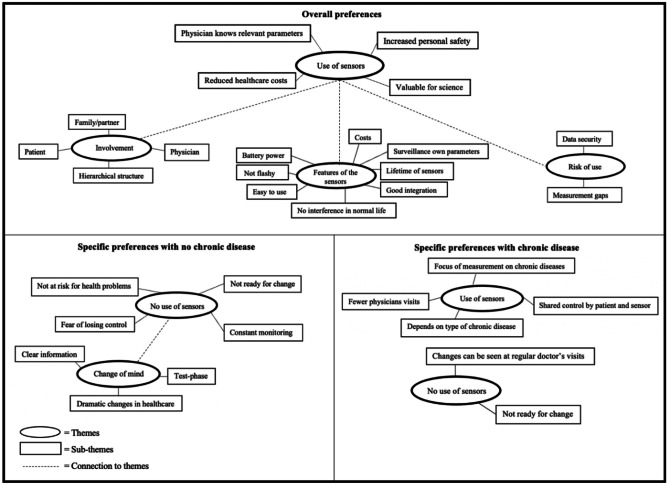


### A.3 Overall preferences - use of sensor

#### Valuable for science

Some interviewees would further use a wearable sensor if it were useful for science. They argued that their information would support research and could be used to improve early detection of disease in other patients. If a large amount of health data were available, better predictions could be made, which would benefit all wearable sensor users.

#### Reduced healthcare costs

A final reason cited for the use of a wearable sensor would be the potential to reduce healthcare spending in Switzerland. As mentioned earlier, the use of a wearable sensor was associated with saving time for patients and doctors. In our society, time is money and thus a large amount of administrative work could be saved. In addition, doctor visits can be reduced to a minimum, if the sensor only detects potential concern that requires further investigation.

### A.4 Overall preferences – features of the sensors

#### Surveillance of own parameters

The possibility of having insight into one’s own parameters was evaluated differently. For some participants it would be “nice to have”, for others an important feature. Those who do not necessarily need to see their own parameters would also only want to see the parameters that interest them or that they have the knowledge to interpret. The desire of self-surveillance lay in the possibility of self-control based on one’s own parameters. This is highly dependent on personal knowledge of the parameters being measured.

“As a nurse, I can estimate quite well what is in the normal range, which is very reassuring for me”.

For people who do not have the specific knowledge, the insight into their own parameters could be rather unsettling.

#### Good integration

One feature that was not necessary for the participants, but would act as a positive side effect, is good integration. Good integration in the sense of integrating the sensor into existing devices or applications. Using the sensor would be more user-friendly if it is compatible with the personal smartwatch, does not require additional gadgets, and can be integrated into existing health applications on the smartphone.

### A.5 Overall preferences – risk of use

#### Measurement gaps

Participants were concerned about measurement gaps during the charging period. What if complications or emergency situations occur exactly during the charging time? Therefore, participants would prefer sensors with long battery life and a fast power supply option to minimize measurement gaps.

### A.6 Overall preferences – involvement

In the interviews, participants were asked about involving others when the sensor detects health complications or deterioration of a chronic diseases. The different preferences are presented below.

#### Patient

Some individuals would prefer to be the only ones to receive notification when the sensor detects a change or complication. These individuals argue that it is their responsibility to contact their primary care physician. People need to stay active and do something for themselves.

#### Family/partner

Other interviewees want a family member or life partner to be involved as well. They see a great advantage in having a few selected close contacts receive notifications as well. These people exert pressure from the outside, can react quickly in emergency situations and have important health related information to organize things if necessary.

#### Primary care physician

In addition, some individuals would like the primary care physician to be actively involved as well. The doctor or his/her assistant could make a call to discuss the current situation and decide whether immediate action is required or whether it is simply necessary to make an appointment.

#### Hierarchical structure

If several people are involved, the participants would prefer a hierarchical structure. Otherwise, everyone is informed, and in the worst case, no one feels responsible for taking actions. Therefore, the involvement should start with those who live near the person wearing the sensor. If there is no response, the notification goes to the second person in line.

### A.7 Specific preferences with chronic disease – use of sensors

#### Fewer physician visits

The fact that the doctor is only seen when needed was valued as a major advantage of wearable sensors in this scenario.

“If I had something now where the doctor says I have to measure a value every two weeks or come by, just regularly every two weeks […] I could imagine that you could turn something like that off with a sensor like that and only go when it’s really necessary.“

If questions came up in the meantime participants would be willing to call their physician instead of making an appointment.

### A.8 Specific preferences without chronic disease – no use of sensors

#### Constant monitoring

One of the main benefits of wearable sensors, constant monitoring, was a feature that was not desired by the participants disliking scenario one. This constant monitoring would be mentally very stressful, as health parameters can be displayed at any time. In addition, constant monitoring would give the false sense that nothing can happen because everything is under control. Participants argued that a wearable sensor cannot measure everything. Even if the sensor were able to record everything, they would dislike the idea of continuous monitoring, since illness is a part of life that must be accepted.

#### Not ready for change

When discussing the reasons for rejecting a wearable sensor, it could be noticed that some interviewees have difficulty accepting change. They had a strong feeling of insecurity that led to rejection. At present, the decision to see a primary care physician is in the hands of the patient, and they feared that this power will be taken away from them. Participants clearly stated that the human interaction is highly valued and there is no desire of changing this interaction to a device. In Fig. 1 there is a link to “no use of sensors” in scenario two, which is also related to “not ready for change.“ A chronic illness is often associated with regular visits to the doctor. These visits were highly valued by the participants, as patients take time to address their illness.

### A.9 Specific preferences without chronic disease – change of mind

Several situations could change the respondents’ opinion from no use to use of a wearable sensor. The discussed situations are highlighted below.

#### Clear information

To move from no use to use of sensors, participants need clear information. They want to know what exactly the circumstances would be. What powers does the primary care physician have with the personal data and what are the patient’s responsibilities. They would need clear studies showing the benefits of such sensors and concrete examples where sensors could prevent serious complications.

#### Test-phase

Some interviewees suffering from type 1 diabetes already wear a sensor to self-monitor their blood glucose levels. They were initially very skeptical about this wearable sensor. Their diabetologist convinced them to try the sensor. After two weeks, they were very satisfied with the sensor. Such a non-binding test phase could also help individuals to become familiar with the wearable sensor presented in this study.

#### Dramatic changes in healthcare

Dramatic changes in healthcare would be a compelling reason to use wearable sensors. The two changes mentioned would be, first, a huge shortage of primary care physicians that would lead to long waiting lists. Second, a dramatic increase in healthcare spending that would make healthcare unaffordable. These two mentioned changes would legitimize the use of wearable sensors.

## Data Availability

Datasets are available on request from the corresponding author, (C. Matti).
